# The effects of Massa Medicata Fermentata on the digestive function and intestinal flora of mice with functional dyspepsia

**DOI:** 10.3389/fphar.2024.1359954

**Published:** 2024-03-01

**Authors:** Shuyu Wang, Yuanlin Li, Xiaoqi Yang, Yinxue Hao, Xueyan Zhan

**Affiliations:** ^1^ School of Chinese Materia Medica, Beijing University of Chinese Medicine, Beijing, China; ^2^ Beijing Key Laboratory for Production Process Control and Quality Evaluation of Traditional Chinese Medicine, Beijing Municipal Science and Technology Commission, Beijing, China

**Keywords:** Massa Medicata Fermentata, intestinal microbiota, 16S rRNA, food accumulation, functional dyspepsia

## Abstract

**Introduction:** The purpose of this study was to identify the chemical components of Massa Medicata Fermentata (MMF) in different fermentation methods, analyze its regulatory effects on gastrointestinal propulsion and intestinal flora in mice with food accumulation, and further explore its mechanism of action in the treatment of dyspepsia.

**Methods:** The chemical compositions of three kinds of MMF were identified using the UPLC-Q- Exactive Orbitrap mass spectrometer. A model of spleen deficiency and food accumulation in mice was established. The gastric emptying rate and intestinal propulsion rate were calculated, serum gastrin concentration and cholinesterase activity were measured, and 16S rRNA microbial detection was performed in different groups of mouse feces.

**Results:** The results showed that a total of 95 chemical components were identified from the three MMF extracts, 62 of which were the same, but there were differences in flavonoids and their glycosides, organic acids, and esters. MMF, PFMMF, and commercial MMF could all significantly improve the gastric emptying rate, intestinal propulsion rate, and GAS concentration in the serum of model mice; PFMMF has a better effect, while there was no significant difference in cholinesterase activity among the groups (*p* > 0.05). The 16S rRNA sequencing results showed that the MMF and PFMMF could increase the content of beneficial bacteria *Bacteroidetes* and decrease the pathogenic bacteria *Verrucomicrobia* in the intestines of model mice, while the commercial MMF could not.

**Discussion:** Studies suggest that MMF has a variety of possible mechanisms for improving food accumulation and treating gastrointestinal dyspepsia, which provides reference value for the quality evaluation and clinical application of MMF.

## 1 Introduction

As we all know, with the improvement of living standards, people pay more attention to health. Functional dyspepsia (FD) is a common functional gastrointestinal disease (Ford et al., 2020) that endangers people’s physical and mental health. Its global incidence rate is 16% (Ford et al., 2020), which is 11%–20% in Western countries and 11.8%–23.8% in China (Aro et al., 2015; Wu et al., 2013), according to a 10-year follow-up study. The pathogenesis of FD is still unclear, but many studies have confirmed that the disease is affected by many factors, such as diet (Göktaş et al., 2016; Hassanzadeh et al., 2016), infection (Gutiérrez et al., 2015; Wensaas et al., 2016), duodenal inflammation (Talley et al., 2007; Wouters et al., 2016; Vanheel et al., 2014), and gastroduodenal dysfunction (Steinsvik et al., 2016). In clinical practice, FD is characterized by slow gastric emptying, impaired fundic relaxation or fundic disaccommodation, and gastroduodenal hypersensitivity to balloon distention (Talley and Ford, 2015; Talley, 2016).

In recent years, researchers have paid attention to traditional Chinese medicine (TCM) to treat functional dyspepsia, which can produce significant recovery results with low side effects (Zhao et al., 2020; Zhang et al., 2019). The fermentation of TCM can enhance its original functions or generate new effects, expanding its application range to meet clinical needs (Zhang et al., 2022). As a fermented TCM widely used in clinical practice, Massa Medicata Fermentata (MMF) is mainly fermented with flour, wheat bran, Vignae semen, Armeniacae semen amarum, Artemisia annua herba, Polygonum hydropiper, and *Xanthium sibiricum*. It is a common medicine for the clinical treatment of functional dyspepsia such as gut microbiota disorder, diet stagnation, or children’s food accumulation (Bai et al., 2023; Liu et al., 2021).

The gut microbiome is one of the most important members of the human body and plays an important role in gastrointestinal health (Futagami et al., 2015). It has been reported that the disturbance of intestinal flora quantity and structure is one of the important causes of FD. Intestinal microorganisms can decompose the gastrointestinal diet into various metabolites and participate in the life activities of the body. Studies have shown that MMF can treat gastrointestinal inflammation, recover the distribution of gut microbiota, and ultimately improve indigestion symptoms. The weaning transition period during piglet growth often leads to stress reactions such as anorexia, growth retardation, severe intestinal infections, and even death (Gresse et al., 2017; Lalles et al., 2007). Dietary supplementation with PFMMF can enhance host intestinal homeostasis by regulating the composition of the gut microbiota and beneficial SCFA levels in piglets, thereby improving this situation (Wang et al., 2018). However, it is not clear how MMF regulates changes in intestinal flora in the treatment of FD.

After preliminary exploration, the research group optimized the preparation process of MMF and fermented MMF by adding *Rhizopus oryzae*. In this study, we evaluated the efficacy of the prepared MMF and PFMMF, and the commercial MMF with the same preparation process as the above two kinds of MMF was evaluated on model mice with food accumulation and gut microbiota disorders. Using commercial MMF as a control, the gastrointestinal propulsion effects and the intestinal homeostasis effects of MMF and probiotic-fermented MMF were evaluated, and gastrin (GAS) and cholinesterase (CHE) were the biochemical indicators used to measure gastrointestinal motility; the abundance of microbial species and every kind of microbiota was detected to evaluate the intestinal homeostasis by 16S rRNA high-throughput sequencing technology. At the same time, the action mechanisms of the prepared MMF and commercial MMF can be preliminarily explored.

## 2 Materials and methods

### 2.1 Reagents

Soluble starch (C10650260) and carboxymethyl cellulose sodium (20190106) were purchased from McLean Biochemical Co., Ltd. (Shanghai, China). D-glucose was purchased from Beijing Baierdi Biotechnology Co., Ltd. (Beijing, China). Activated carbon was purchased from Tianjin Guangfu Technology Development Co., Ltd. (Tianjin, China). Fish floss (20220601) was purchased from Xiamen Xiashang Huangjinxiang Food Co., Ltd. (Fujian, China). Bean powder (20220611) was purchased from Heilongjiang Province Beidahuang Green Health Food Co., Ltd. (Heilongjiang, China). Flour (BXT20211022) was purchased from Jinshahe Noodle Industry Group Co., Ltd. (Hebei, China). Milk powder (647FG 1905) was purchased from Inner Mongolia Yili Industrial Group Co., Ltd. (Inner Mongolia Autonomous Region, China). MS-grade formic acid (212271), methanol (221713), and acetonitrile (224288) were purchased from Thermo Fisher Scientific (China) Co., Ltd.

### 2.2 Preparation of MMF

On the basis of the MMF prescription in Volume 19 of the “Drug Standards of the Ministry of Health of the People’s Republic of China-Traditional Chinese Medicine Formulas” (Chinese Pharmacopoeia Committee, 1998), soft materials for subsequent fermentation were prepared, then *R. oryzae* seed liquid was added and compacted into a cuboid, and the cuboid was fermented under a constant temperature and humidity to make koji. Simultaneously, MMF without adding *R. oryzae* seed liquid was prepared as a control. The commercial MMF was purchased from a traditional Chinese medicine company in Sichuan Province.

The appropriate amount of MMF sample was taken, four times the quantity of water was added, mixed evenly, sonicated at 40°C for 1 h, and centrifuged at 8,000 rpm for 10 min. The supernatant was concentrated to the required concentration and then administered to mice by gavage.

After conducting preliminary experiments on the extraction solvents (methanol and water), it was found that the chemical components were mainly enriched in methanol solvents. Based on the chromatogram, methanol was ultimately selected as the extraction solvent. A measure of 7.5 g of MMF was accurately weighed, then 40 mL of methanol was added, ultrasounded at 40°C for 1 h, and centrifuged (12,000 r min^-1^ × 10 min), and 20 mL of supernatant was taken and dried by evaporation. Methanol was added to the residue, the constant volume of which was 10 mL, and the test solution was obtained through the 0.22 μm microporous filtration membrane.

### 2.3 Chemical composition analysis of MMF with different fermentation methods

#### 2.3.1 Analytical conditions for UPLC-Q-Exactive Orbitrap MS

A measure of 7.5 g of MMF was accurately weighed, 40 mL of methanol was added, ultrasounded at 40°C for 1 h, and centrifuged (12,000 r·min^-1^ × 10 min), and 20 mL of supernatant was taken and dried by evaporation. Methanol was added to dissolve the residue and fixed at a constant volume of 10 mL; then, the test solution was obtained through the 0.22 μm microporous filtration membrane.

The chemical components in MMF were qualitatively analyzed by the UPLC-Q-Exactive Orbitrap MS technique. A UPLC-Q-Exactive Orbitrap MS (Thermo Fisher Scientific (China) Co., Ltd., model: Ultimate 3000) was used for mass spectrometry analysis. XSelect HSS T3 (4.6 × 250 mm, 5 μm; Waters) was used for sample separation. The column temperature was 35°C. The mobile phase was 0.1% formic acid (A) and acetonitrile (B), with a flow rate of 0.3 mL/min. The gradient elution procedure was as follows: 0–5 min, 95%–90% A; 5–15 min, 90%–80% A; 15–22 min, 80%–75% A; 22–32 min, 75%–65% A; 32–42 min, 65%–5% A; 42–50 min, 5% A; 50–55 min, 5%–95% A; and 55–60 min, 95% A. The injection volume was 5 μL. In addition, the mass spectrometer conditions under positive and negative ion modes were as follows: the spray voltages were (+) 3800 v and (−) 3200 v; the sheath gas flow rate and auxiliary gas flow rate were 1.05 × 10^4^ mL/min and 4.5 × 10^3^ mL/min, respectively; the temperature of the ion transfer tube was 350°C; the temperature of the auxiliary gas was 300°C; and the collision energies of MS2 were 30 V, 40 V, and 50 V. In addition, the range of data collection was 100–1500 m/z with a full scan.

#### 2.3.2 Chemical compositions in MMF identification

The names, structural types, molecular formulas, theoretical mass, measured mass, and main fragments of the chemical constituents in MMF and its raw materials were sorted out, and the mass spectrum database of the chemical constituents in MMF was established. Xcalibur 4.0 software was used to calculate the theoretical mass value of the chemical components, extract the measured mass of the corresponding chemical components, and compare the measured mass with the above theoretical mass, the measured main fragment, and the reported main fragment. Then, the possible cleavage pathway of the main fragment was predicted according to the ions of the main MS fragment, and the chemical components in MMF were further identified.

### 2.4 Effects of MMF on digestive function in mice

#### 2.4.1 Animals and model animals

Thirty healthy SPF-grade ICR male mice weighing 18–22 g were purchased from Sperford (Beijing) Biotechnology Co., Ltd. All animals were kept at a temperature of (24 ± 2)°C, a humidity of (60 ± 5) %, and a light and dark period of 12/12 h. All experimental procedures involving animals were approved by the Animal Ethics Committee of the Beijing University of Chinese Medicine Animal Laboratory Center (Ethical Review Approval No. BUCM-4-2022110702-4027).

Thirty male ICR mice were randomly divided into five groups, with six mice in each group. The groups were divided into the normal control group (Con), model group (Mod), naturally fermented MMF without *R. oryzae* administration group (MZ, 4 g kg^-1^), naturally fermented MMF with *R. oryzae* administration group (M, 4 g kg^-1^), and commercial MMF administration group (S, 4 g kg^-1^) and were fed adaptively for 3 days. Then, the model group and three drug administration groups were fed with self-made high-protein and high-calorie feed and 50% milk (0.02 mL g^-1^ body weight), while the normal control group was fed with conventional mice feed and an equal dose of physiological saline for 7 consecutive days. The general behavior, body weight, food intake, water intake, and fecal volume of each group were observed every day. Compared with the normal control group, the body weight of the mice in the model group and the three-drug administration groups increased slowly, the water intake increased, and the food intake and fecal volume decreased. The model mice showed restlessness and even fighting, abdominal obesity, brown-red feces, and thick texture, which indicated that the model mice had the symptoms of food accumulation (Zhang et al., 2020).

After the successful modeling of the mice with spleen deficiency and food accumulation, the three-drug administration groups were given continuous intragastric administration for 7 days, while the normal control group and the model group were given the same amount of normal saline until the end of the experiment.

#### 2.4.2 Measurement of the gastrointestinal motility index

The gastrointestinal motility of model mice was characterized by the gastric emptying rate and small intestine propulsion rate.

A measure of 10 g of sodium carboxymethyl cellulose was weighed into a beaker, and 250 mL of water was added and stirred until fully dissolved. A measure of 16 g of milk powder, 8 g of D-glucose, and 8 g of activated carbon were added successively to make a black semi-solid paste, which was used for gastric emptying and small intestine propulsion experiments.

Each group was dehydrated and fasted for 18 h before administration. 0.5 h after the last administration, each group was gavaged 0.8 mL of carbon powder semi-solid paste. After 15 min, the eyeballs were taken, and the blood was centrifuged at 3,000 rpm for 15 min. The serum was collected and stored in the refrigerator at −80°C for detection. After taking the blood, the mice were killed, and the whole stomachs were taken. After wiping with filter paper, the total weight of the stomach was measured, and the data were recorded. The stomach contents were rinsed with normal saline and then dried with filter paper. The net weight of the stomach was measured and recorded. The gastric emptying rate was calculated according to the following equation (Liu et al., 2023a):

$$\text{gastric emptying rate}\left(\%\right)=\left(1‐\frac{W_{1}‐W_{2}}{W_{3}}\right)\times 100\%.$$ (1)

W_1_ is the total weight of the stomach, W_2_ is the net weight of the stomach, and W_3_ is the quality of 0.8 mL of carbon powder semi-solid paste.

After dissecting the mice, the intact small intestine was taken, straightened, and placed on a piece of smooth white paper. The total length of the small intestine was measured, and the data were recorded. The length from the front of the small intestine to the front of the carbon powder was measured as the push length of the carbon powder semi-solid paste, and the data were recorded. The intestinal propulsion rate was calculated according to the following equation (Liu et al., 2023b):

$$\text{small intestine propulsion rate}\left(\%\right)=\frac{L_{1}}{L_{2}}\times 100\%.$$ (2)

L_1_ is the pushing length of the carbon powder semi-solid paste; L_2_ is the total length of the small intestine.

#### 2.4.3 ELISA assays

Gastrin (GAS) can improve gastrointestinal hormone secretion and increase gastrointestinal motility. Cholinesterase (CHE) stimulates the gastrointestinal smooth muscle to contract. Gastrin (GAS) concentration (Yuanju Biotechnology, Shanghai, China) and cholinesterase (CHE) activity (Jiancheng Bioengineering Institute, Nanjing, China) were measured according to the kit instructions.

#### 2.4.4 16S rRNA sequencing assay

The 16S rRNA technology was used to detect fecal microorganisms in mice with food accumulation before and after administration. After the total DNA was extracted from fecal samples of different groups of mice, specific primers were synthesized according to the full-length primer sequence, namely, forward primer 5′-cctacgggnggcwggag-3′ and reverse primer 5′-GACTACHVGGGTATCTAATCC-3,′ and the V3 and V4 regions of the 16S rRNA gene were amplified. The first round of amplification products was purified as the template for the second round of PCR amplification, and the amplified products were purified, quantified, and homogenized to form a sequencing library. The quality of established libraries was first checked, and qualified libraries were sequenced using Illumina MiSeq. After the sequencing data were preprocessed, bioinformatics analysis was performed.

### 2.5 Data analysis

The drawing was completed using GraphPad Prism 9.4.1 software (GraphPad Software Inc., San Diego, California, United States). SPSS (Social Science Statistics Package) V26 software (SPSS Inc., Chicago, IL, United States) was used to analyze the data. *p* < 0.05 was considered statistically significant.

There was a certain proportion of dirty data in the original data obtained by sorting. In order to make the results of the information analysis more accurate and reliable, the original data were first spliced and filtered to obtain effective data. The merge sequence was obtained by using the fastq_mergepairs command in vsearch. Cutadapt software was used to remove primers from the sequence. The vsearch’s fastq_filter command was used to delete low-quality sequences, deleted sequences containing N bases, and deleted sequences less than 100 bp in length. The clean data were then used for operational taxonomic clustering and species taxonomic analysis. Sequences with 97% similarity were clustered, and sequences with similarity greater than 97% were clustered into the same OTU. The representative OTU sequences were selected to be compared with the database for OTU classification labeling, and the species information with the highest similarity to the OTU sequences with more than 80% confidence was used for OTU labeling.

## 3 Results

### 3.1 Chemical compositions in MMF with different fermentation methods

**FIGURE 1:**
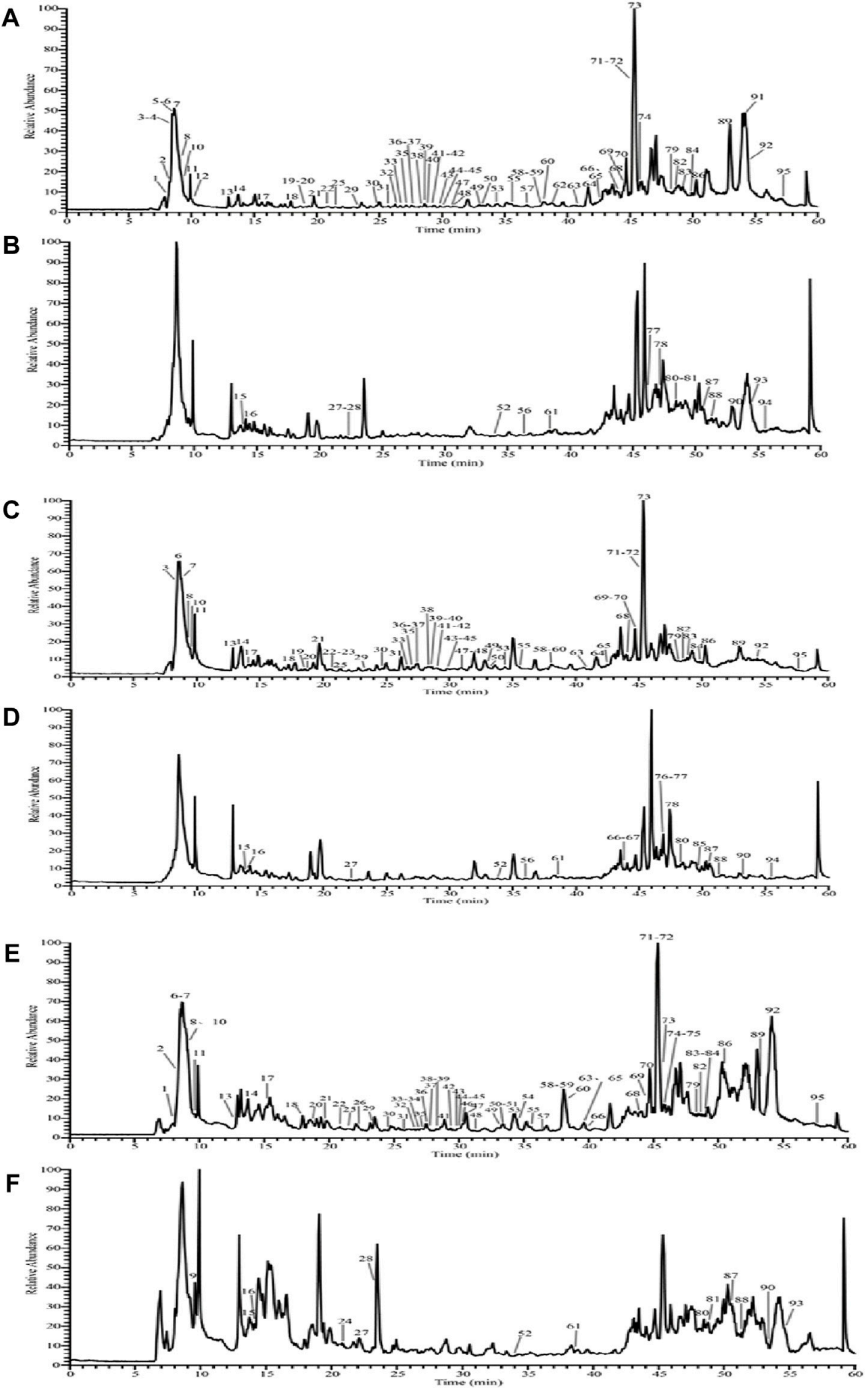
Total ion flow diagram of MMF with three different fermentation methods. Negative ion mode total ion flow diagram of the naturally fermented MMF without *R. oryzae* **(A)**; positive ion mode total ion flow diagram of the naturally fermented MMF without *R. oryzae* **(B)**; negative ion mode total ion flow diagram of the naturally fermented MMF with *R. oryzae* **(C)**; positive ion mode total ion flow diagram of the naturally fermented MMF with *R. oryzae* **(D)**; negative ion mode total ion flow diagram of the commercial MMF **(E)**; positive ion mode total ion flow diagram of the commercial MMF **(F)** (each serial number represents the presence of a chemical component).

As shown in Figure 1, the total ion flow graphs of MMF without adding *R. oryzae* (Figures 1A, B), MMF naturally fermented with *R. oryzae* (Figures 1C, D), and commercial MMF (Figures 1E, F) in positive and negative ion modes are shown, respectively. The compound fragment information of MMF was obtained by using a UPLC-Q-Exactive Orbitrap-MS, and the relevant high-resolution mass spectrometry data of the chemical components contained in MMF and its raw materials were sorted out, combined with literature reports, and Supplementary Table S1 was obtained.

The experimental results showed that a total of 95 components were identified from the MMF of three different fermentation methods (Figures 1A–F; Supplementary Table S1). There were 62 common chemical components in the three kinds of MMF, including 14 flavonoids and their glycosides, 25 organic acids and their esters, 4 carbohydrates, 9 phenylpropanoids, 2 amino acids, 2 nucleotides, 2 alkaloids, 1 vitamin, 1 hydrocarbon, and 2 phenolics. Comparative analysis showed that the chemical composition types of MMF obtained by different fermentation methods were not exactly the same. For example, terpenoids and acid anhydrides were identified in PFMMF. The former was not identified in MMF or commercial MMF, while the latter was not identified in commercial MMF. In addition, PFMMF differed from MMF and commercial MMF mainly in flavonoids and glycosides, organic acids, and ester compounds, which were mainly related to the inoculation of *R. oryzae* during the fermentation process. Comparing Figures 1A–D, it was found that in the total ion flow pattern of mass spectrometry during the period of 15–35 min, the relative response values of 12 chromatographic peaks for qualitative identification of MMF as organic acids without adding *R. oryzae* were 2%–8%, while the relative response values of 13 chromatographic peaks for qualitative identification of MMF as organic acids by inoculation of *R. oryzae* were 4%–22%. It was suggested that *R. oryzae* inoculates and ferments MMF to produce more organic acids, and organic acid compounds can promote intestinal digestion and absorption and regulate intestinal microorganisms (He et al., 2020; Wu and Sun, 2009; Zhang et al., 2013).

### 3.2 Effects of MMF on the gastrointestinal motility of model mice with food accumulation

**FIGURE 2:**
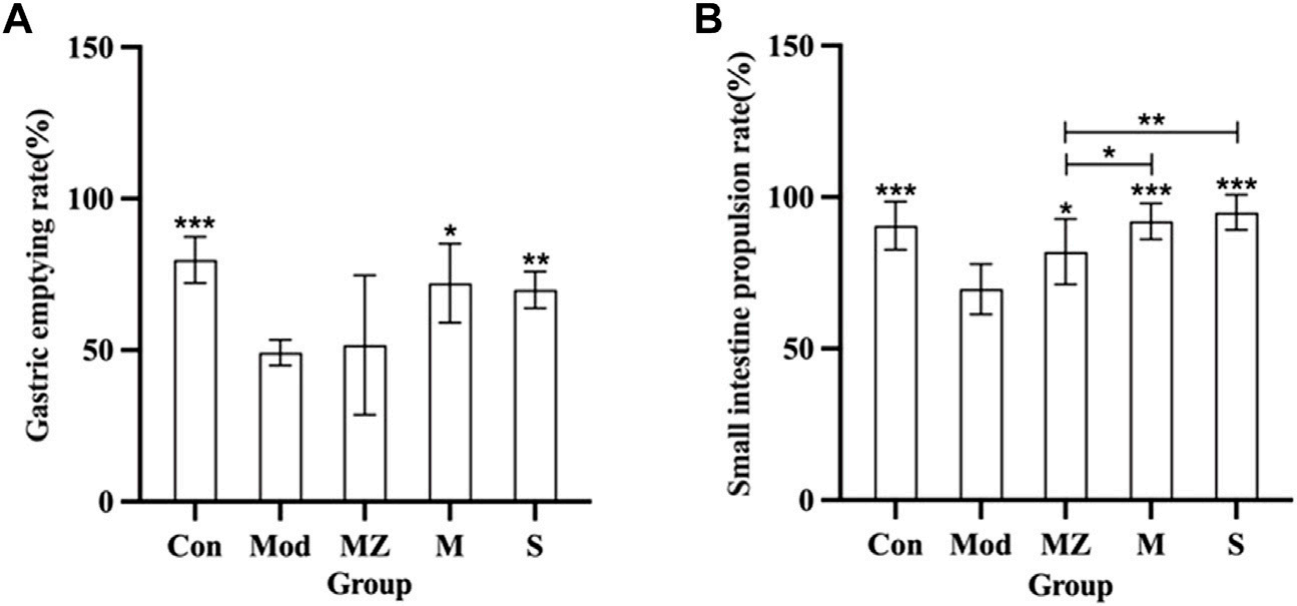
Measurement results of the gastric emptying rate and intestinal propulsion rate in different treatment groups (*n* = 6). Effects of different treatment groups on the gastric emptying rate in mice **(A)**; effects of different treatment groups on small intestine propulsion rate in mice **(B)**. Compared with the model group, *p < 0.05, **p < 0.01, and ***p < 0.001.

From Figure 2, it can be seen that, compared with the normal control group, the gastric emptying rate and small intestine propulsion rate of the model group mice were significantly reduced (*p* < 0.001), indicating that the food accumulation model was successfully established. Compared with the commercial MMF administration group, naturally fermented MMF and PFMMF significantly improved the gastric emptying rate (*p* < 0.05, *p* < 0.01), and small intestine propulsion rate (*p* < 0.001, *p* < 0.001) of model mice with food accumulation, respectively. This indicates that both MMF and PFMMF can enhance the gastrointestinal motility of model mice. In addition, compared with the MMF administration group, the small intestine propulsion rate of the PFMMF administration group was significantly improved (*p* < 0.05), indicating that the improvement of the small intestine propulsion rate of model mice by the PFMMF fermented with *R. oryzae* may be related to the inoculation of the dominant fungus *R. oryzae* during the fermentation process.

### 3.3 Effects of MMF on GAS and CHE concentrations in the serum of model mice

**FIGURE 3:**
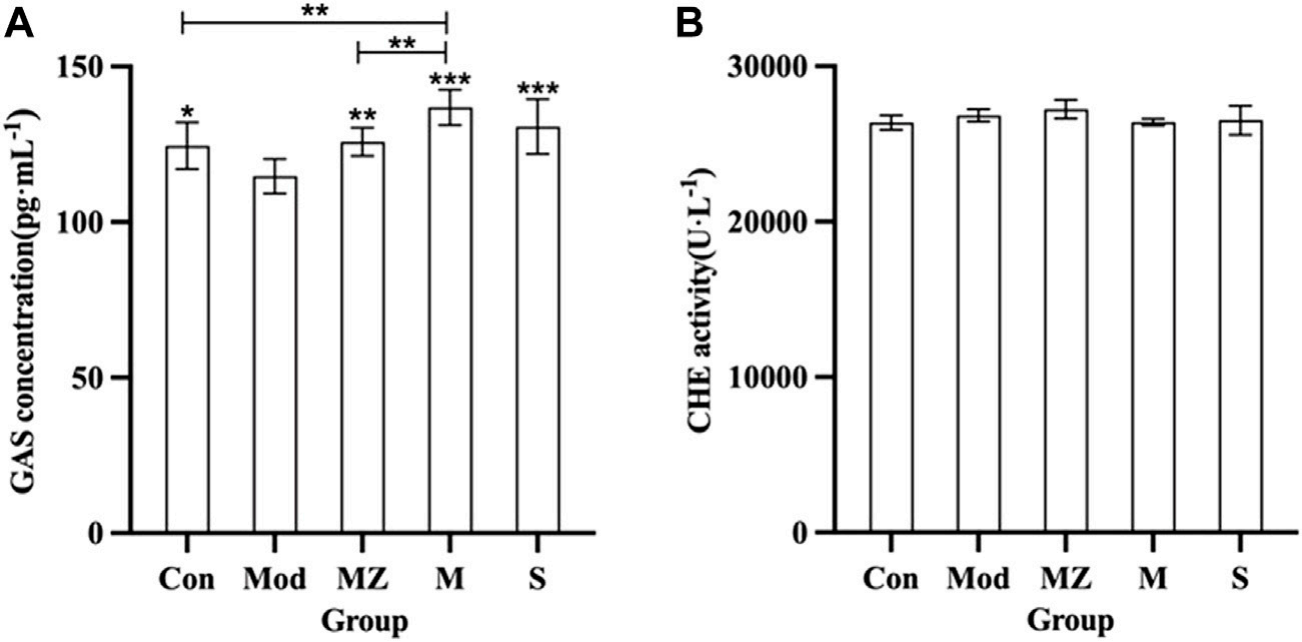
Measurement results on GAS concentration and CHE activity in the serum of mice in different treatment groups (*n* = 6). Effects on serum GAS concentration in different treatment groups **(A)**; effects on serum CHE activity of mice in different treatment groups **(B)**. Compared with the model group, *p < 0.05, **p < 0.01, and ***p < 0.001.

Figure 3 shows that the GAS concentration in the serum of the model group mice decreased significantly (*p* < 0.05) compared with the normal control group. Compared with the model group, GAS concentration in the serum of model mice in the MZ, M, and S groups was significantly increased (*p* < 0.01, *p* < 0.001, and *p* < 0.001), respectively, indicating that the three kinds of MMF could positively regulate GAS concentration in the serum of model mice with food accumulation. The concentration of GAS in the serum of model mice treated with PFMMF was higher than that of the normal control group (*p* < 0.01) and the MMF administration group (*p* < 0.01), indicating that PFMMF fermented with *R. oryzae* had a better upregulation effect on GAS concentration in the serum of model mice. However, there was no significant difference among the five groups (*p* > 0.05) for CHE concentration in the serum of mice.

### 3.4 Effects of MMF on the gut microbial structure and abundance of model mice

#### 3.4.1 Diversity analysis of gut microbial species

**FIGURE 4:**
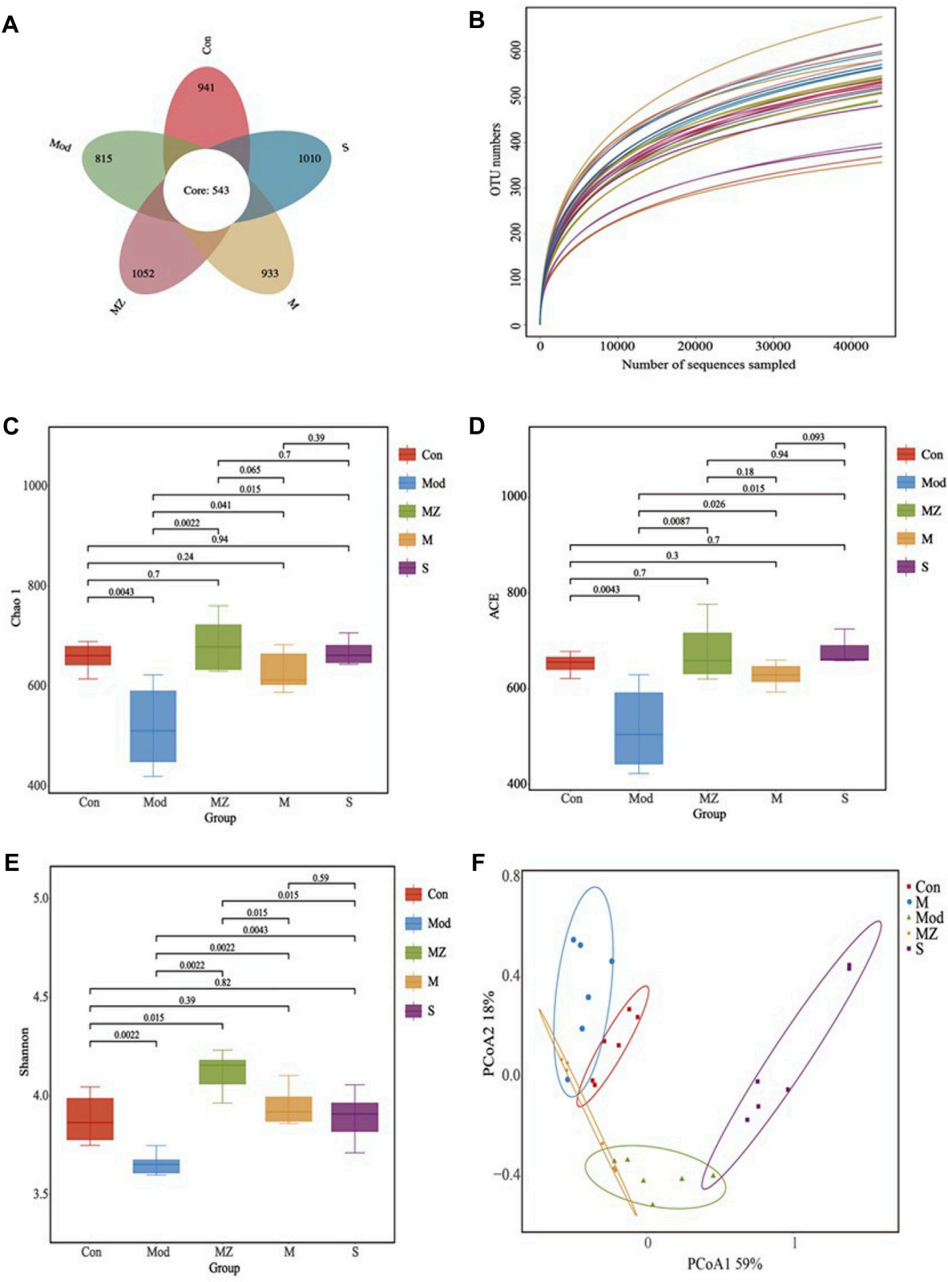
Analysis results of intestinal microbial species diversity in different treatment groups (*n* = 6). Petal diagram showing the number of OTUs in different treatment groups **(A)**; the dilution curve reflects the sample size of different treatment groups **(B)**; Chao 1 index reflecting species richness in different treatment groups **(C)**; ACE index reflecting species diversity and stability in different treatment groups **(D)**; Shannon index reflecting species diversity in different treatment groups **(E)**; and PCoA analysis reflecting species abundance and similarity in different treatment groups **(F)**.

Using the petal plot, dilution curve, Chao 1 index, ACE index, Shannon index, and PCoA analysis as the evaluation index, the effects of MMF, PFMMF, and commercial MMF fermented with three different methods on the diversity and abundance of gut microbiota in food accumulation model mice were investigated (Figure 4).

The high-quality sequence cluster of 30 samples had a total of 543 OTUs at the 97% similarity level. The OTU values of the normal control group, model group, MMF administration group, PFMMF administration group, and commercial MMF administration group were 941, 815, 1052, 933, and 1010, respectively (Figure 4A). The dilution curves all tended to be flat, indicating that the sequencing data volume was reasonable and sufficient to cover all class groups (Figure 4B). In a diversity analysis of intestinal flora, the Chao 1 index and ACE index can reflect the abundance of gut flora, while the Shannon index can reflect the diversity of intestinal flora. The Chao 1 index (Figure 4C), ACE index (Figure 4D), and Shannon index (Figure 4E) of the model group were significantly lower than those of the normal control group (*p* < 0.01), indicating that the abundance and diversity of the gut microbiota in the model group were significantly lower than those in the normal group. However, three MMF administration groups could reverse the above situation, and the species and distribution uniformity of gut microbiota increased after the model group was treated with three kinds of MMF. Compared with the normal control group, there was no significant difference (*p* > 0.05) in the Chao 1 index, ACE index, and Shannon index of the PFMMF administration group, indicating that after treatment with the PFMMF with *R. oryzae*, the types and distribution uniformity of intestinal flora in model mice were similar to those of normal mice. However, compared with the MMF administration group, there was no significant difference (*p* > 0.05) in the Chao 1 index and ACE index of the PFMMF administration group, while there was a significant decrease (*p* < 0.05) in the Shannon index, which indicated that during the natural fermentation process of MMF, the addition of *R. oryzae* increased the proportion of *R. oryzae* and its metabolites in MMF, resulting in a decrease in the uniformity of gut microbial distribution (Figures 4C–E).

The PCoA analysis in intestinal flora *β* diversity analysis not only reflects the gut microbial species composition and abundance but also combines species evolution relationships to comprehensively evaluate the samples’ similarity. The PCoA analysis results showed that the total variance percentages explained by PCoA1 and PCoA2 were, respectively, 59% and 18%, and the normal control group, model group, and three treatment groups were well separated at the level of 77% of the total variance explained by the first two PCoAs. There was a significant difference in the intestinal microbial structure between model mice and normal mice. After model mice were treated with MMF or PFMMF, the intestinal microbial structure of mice underwent changes and was similar to that of normal mice (Figure 4F).

#### 3.4.2 Effects of MMF on gut microbial structure and abundance

In order to visually display the gut microbiota structure of different experimental groups, the top 10 species with abundance in phylum and genus levels were selected to make stacked bar charts, and the changes in gut microbial structure and abundance were observed among the groups.

**FIGURE 5:**
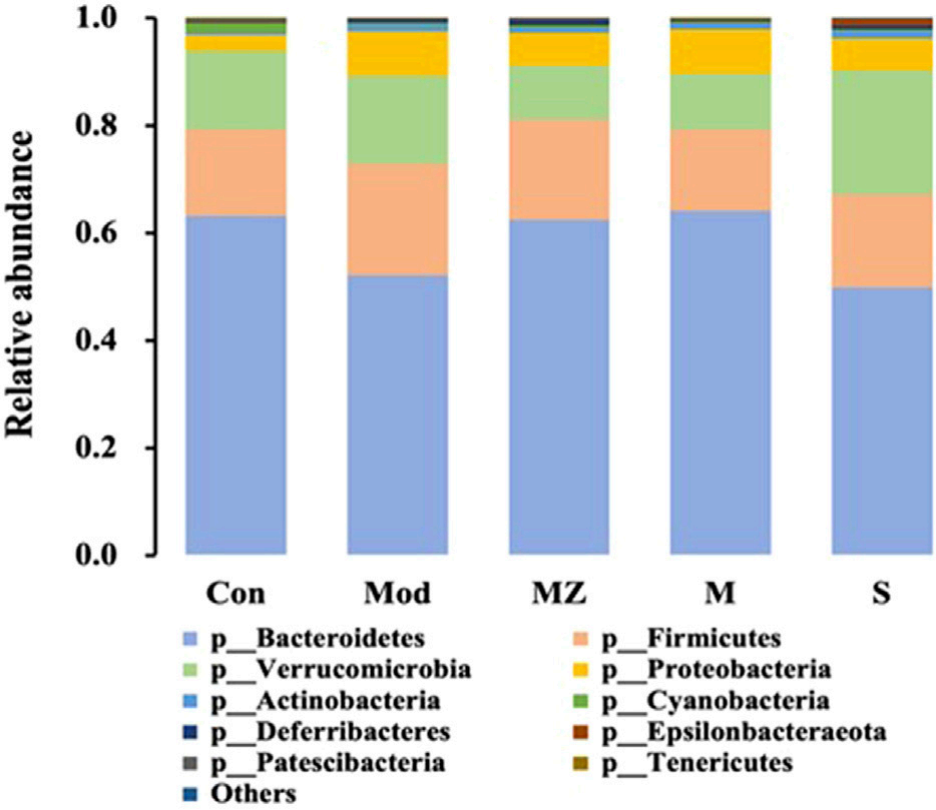
Analysis of the relative abundance of intestinal flora in different treatment groups at the gate level (*n* = 6).

At the phylum level, the five groups were mainly composed of *Bacteroidetes*, *Firmicutes*, *Verrucomicrobia*, and *Proteobacteria*, with a total proportion of more than 96%. Compared with the normal control group, the content of *Bacteroidetes*, a kind of beneficial bacteria, in the intestinal microbiota of the model group mice decreased from 63.2% to 52.1%. The content in the MMF administration group, PFMMF administration group, and commercial MMF administration group was 62.4%, 64.1%, and 49.9%, respectively. On the other hand, compared with the normal control group, the content of conditional pathogenic bacteria *Verrucomicrobia* in the intestinal microbiota of the model group mice increased from 14.6% to 16.4%, and the content of the three treatment groups was 10.0%, 10.4%, and 23.1%, respectively (Figure 5). It could be seen that self-made MMF could adjust the intestinal microbial structure of model group mice, making it tend to normal.

**FIGURE 6:**
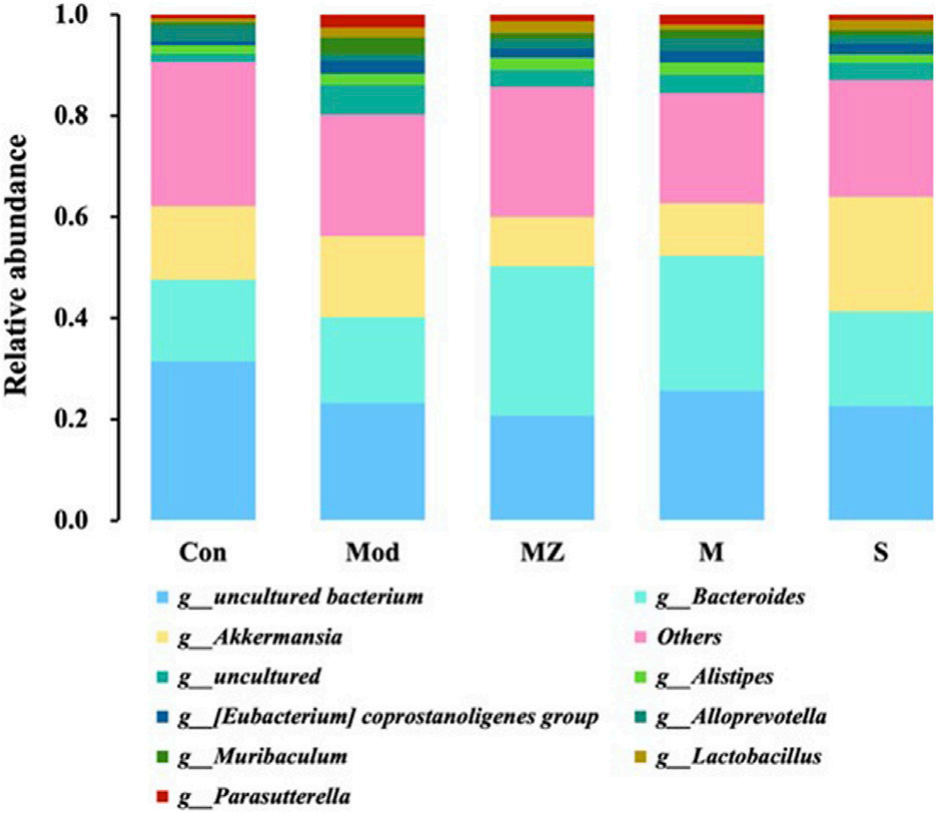
Analysis of the relative abundance of intestinal flora in different treatment groups at the genus level (*n* = 6).

At the genus level, the five groups mainly consisted of *Bacteroides* ***,*** *Akkermansia*, *Alistipes*, *Eubacterium coprostanoligenes group*, *Alloprevotella*, *Muribaculum*, *Lactobacillus*, and *Parasutterella*. Compared with the normal control group, the content of beneficial bacteria *Alloprevotella* in the intestinal microbiota of mice in the model group decreased from 3.0% to 1.2%, while the content in the MMF group, PFMMF group, and commercial MMF administration group was 1.7%, 2.4%, and 1.4%, respectively. The content of beneficial bacteria *Bacteroides* in the normal control group and model group was 16%, while the content in the three treatment groups was 29%, 27%, and 18%, respectively. Compared with the normal control group, the content of *Akkermansia* in the intestinal microbiota of the model group mice increased from 15% to 16%, and the content in the three treatment groups was 9.8%, 10%, and 23%, respectively (Figure 6). The contents of the pathogenic bacteria *Muribaculum* and *Parasutterella* were 0.76% and 0.75% in the normal control group and 3.4% and 2.6% in the model group, respectively. After administration, the microflora content of the three treatment groups changed by 1.2%, 1.8%, and 1.2%, and 1.3%, 2.0%, and 1.1%. It could be seen that self-made MMF could adjust the intestinal microbiota structure of model group mice, making it tend to normal, which was consistent with the changes in intestinal microbiota structure at the phylum level.

**FIGURE 7:**
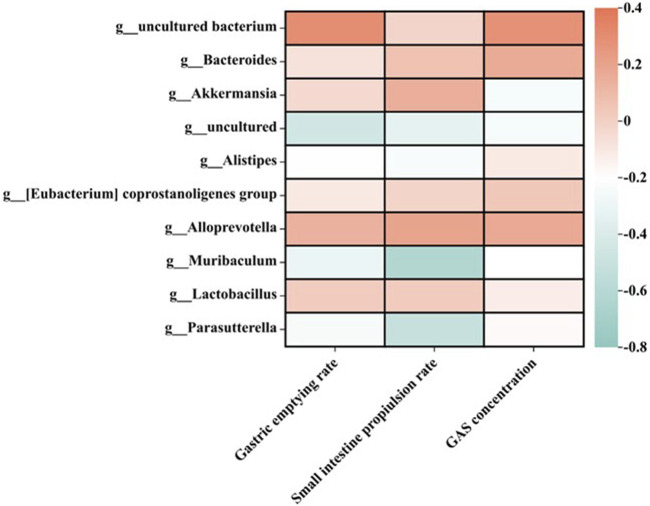
Relationship between gastric emptying rate, intestinal propulsion rate, and GAS concentration in different treatment groups at the genus level (*n* = 6).

The intestinal microbiota is closely related to gastrointestinal function (Wang et al., 2019), and the homeostasis of intestinal microbiota plays an important role in the treatment of indigestion (Agah et al., 2020). Based on the above data, the correlation between intestinal flora and gastrointestinal motility was analyzed. The relationship between 10 high-abundance genera of gut microbiota and gastric emptying rate, intestinal propulsion rate, or gastrin concentration is shown in Figure 7. It was found that gastric emptying rate, intestinal propulsion rate, and GAS concentration were all positively correlated with *Bacteroidota, Bacteroides,* and *Alloprevotella*. In addition, the small-intestine propulsion rate showed a negative correlation with *Muribaculum* and *Parasutterella*.

In terms of the effects of the MMF administration on the intestinal microbial structure of mice, compared with the normal control group at the phylum and genus level, the content of beneficial bacteria *Bacteroidetes* decreased in the intestinal microflora of mice in the model group, while the content of the conditional pathogen *Verrucomicrobia* increased. After the self-made MMF treatment, the content of beneficial bacteria *Bacteroidetes* increased in the intestinal microflora of the model group, while the content of *Verrucomicrobia* decreased, and the intestinal microflora structure tended to be normal. In other words, in the model group, the contents of beneficial bacteria *Bacteroidetes* and *Alloprevotella* decreased, and the gastric emptying rate, intestinal propulsion rate, and GAS secretion decreased, while the contents of conditional pathogenic bacteria *Muribaculum* and *Parasutterella* increased, and the intestinal propulsion rate increased. Gastrointestinal function improved after treatment. It was worth noting that at the phylum and genus level, the ratio of beneficial bacteria and opportunistic bacteria after treatment in the MMF commercial administration group was closer to that in the model group, which was consistent with the results of PCoA analysis.

## 4 Discussion

In this study, a mouse model of spleen deficiency and food accumulation was established. The pharmacodynamics of natural fermentation of MMF without adding *R. oryzae*, MMF inoculated by *R. oryzae*, and commercial MMF were evaluated from three aspects: gastrointestinal motility, serum index, and intestinal microbial structure. The digestion-promoting activity of MMF under different fermentation methods was discussed, and its mechanism was discussed. The results showed that the self-made MMF with *R. oryzae* was better than the other two kinds of MMF in promoting gastrointestinal motility, regulating the structure of intestinal microorganisms, and playing a role in treating dyspepsia.

It is generally believed that the pathogenesis of FD may include increased gastric emptying time, gastrointestinal motility disorders, *Helicobacter pylori* infection, and depression. The use of chemical agents alone in the treatment of FD still has some limitations. The use of traditional Chinese medicine in the treatment of FD has a long history and great potential. TCM can play a role in regulating brain–intestinal peptides, regulating gastrointestinal hormone content, improving stomach peristalsis, regulating immune function, regulating intestinal bacteria, regulating inflammation, etc. (Liu et al., 2023b). Studies have shown that *Aurantii fructus* can increase the secretion of motilin, gastrin, and vasoactive peptide substances, relieve gastrointestinal motility disorders, regulate intestinal flora in rats with dyspepsia, promote the growth of beneficial bacteria, and thus treat FD (Lin et al., 2012; Zhang and Li, 2018). *Hordei Fructus Germinatus* has the function of soothing the liver, nourishing the spleen, and appetizing. Stir-fried *Hordei Fructus Germinatus* can increase gastric secretion and regulate gastrointestinal function (Tian, 2017). The Chinese herb *Aucklandiae Radix* can accelerate gastric emptying rate and motilin release and improve FD symptoms (Chen et al., 1994; Ma, 2021). Under appropriate conditions, the fermentation of TCM under the action of microorganisms has become one of the methods of TCM processing. The fermentation process of TCM through microorganisms can promote the transformation of active ingredients, thus enhancing its efficacy, promoting absorption, and reducing toxicity (Ai et al., 2019; Calvo-Lerma et al., 2022). For example, probiotic fermentation of ginseng significantly increases ginsenoside content, stimulates anti-inflammatory, antioxidant, and immune activity, and can also regulate intestinal flora and immune function to alleviate diarrhea symptoms (Qu et al., 2021). The MMF is made by mixing Semen Armeniacae Amarum (SAA), Semen Vignae (SV), flour, and wheat bran (WB) with water extracts of Herba Artemisiae Annuae (HAA), Herba Polygoni (HP), and Herba Xanthii (HX) in a certain proportion (Ren and Song, 2010) and has the effect of digestion, strengthening the spleen, warming the stomach, and promoting digestion. This research group optimized the fermentation process of MMF in the early stages and proposed that the dominant bacteria were inoculated in the natural fermentation process of MMF, the quality of MMF was improved after the addition of *R. oryzae*, and the digestive promotion effect of MMF after the addition of *R. oryzae* was stronger than that without *R. oryzae* and commercial MMF.

Gastrin is a hormone that exists in the gastrointestinal tract and improves its secretion function, which can increase gastrointestinal motility. Cholinesterase, as an excitatory neurotransmitter of gastrointestinal motility, can excite gastrointestinal smooth muscle to make it contract. In this study, the GAS level of mice in the MZ group, M group, and S group was significantly increased after three kinds of MMF treatment, and the serum GAS concentration of mice treated with PFMMF was higher than that of the normal control group (*p* < 0.01) and MMF administration group (*p* < 0.01), indicating that the addition of PFMMF fermented by *R. oryzae* had a better upregulation effect on the concentration of GAS in the serum of dyspeptic mice.

The imbalance of intestinal flora is closely related to FD, and the regulation of intestinal microbial structure is one of the most effective ways to treat dyspepsia. *Bacteroidetes* and *Firmicutes* are dominant bacteria in the human gut (Liu et al., 2021). In order to investigate whether the improvement of FD by fermentation of MMF is related to changes in intestinal flora, 16S rRNA sequencing was used to study the effect of MMF on intestinal microorganisms in mice with splenic deficiency and food accumulation. The results showed that the intestinal microbial structure of the mice with spleen deficiency and food accumulation was significantly disordered compared with the normal group, and the intestinal microbial structure tended to be balanced after the treatment of MMF, which was close to the state of normal mice. We also found that the content of beneficial bacteria *Bacteroides* decreased and the content of harmful bacteria *Verrucomicrobia* increased in the model group, which was reversed after treatment with MMF. In addition, beneficial bacteria *Bacteroides* were positively correlated with gastrin content (Zhang et al., 2020). Self-made MMF significantly increased the content of beneficial bacteria in the gut and serum gastrin content in mice with splenic deficiency and food accumulation. We speculated that MMF can regulate the intestinal microbial structure and then affect the levels of SCFAs and other metabolites produced by the flora so as to maintain a balanced gastrointestinal environment and treat dyspepsia. However, the effects of self-made MMF and commercial MMF on the metabolites of intestinal flora need to be further verified.

In summary, the chemical components of the three MMFs were highly similar, and pharmacological experiments and analysis of gut microbiota based on 16S rRNA technology were used to study the efficacy and mechanism of action of MMF with different fermentation methods. Our research results indicated that self-made MMF could adjust the intestinal microbial structure of model group mice, making it closer to the normal control group. Among them, beneficial bacteria *Bacteroidetes* were positively related to the secretion of GAS. In addition, commercial MMF could not only increase the content of beneficial bacteria *Bacteroidetes* but could also promote the secretion of GAS to increase gastrointestinal motility. The authors showed that the addition of *R. oryzae* after fermentation process optimization changed the structure of the bacteria contained in the self-made MMF, thus promoting the change in GAS secretion and intestinal flora, suggesting that the regulation of intestinal microbial structure is one of the mechanisms of MMF in the treatment of spleen deficiency and food retention. This study provides a reference value for the quality evaluation and clinical application of fermented MMF.

## Data Availability

The data presented in the study are deposited in the NCBI repository, accession number PRJNA1068858.

## Abbreviations

FD, functional dyspepsia; MMF, Massa Medicata Fermentata; PFMMF, probiotics-fermented MMF; Con, control group; Mod, model group; MZ, natural fermentation MMF group without adding *R. oryzae*; M, natural fermentation MMF group with adding *R. oryzae*; S, commercial MMF administration group; GAS, gastrin; CHE, cholinesterase; OUT, operational taxonomic units; PCoA, principal co-ordinates analysis; and SCFAs, short-chain fatty acids.
