# Supplementary material for: The effects of Massa Medicata Fermentata on the digestive function and intestinal flora of mice with functional dyspepsia
Source: Front Pharmacol. 2024 Mar 1;15:1359954. doi: 10.3389/fphar.2024.1359954 (PMC10941201; doi:10.3389/fphar.2024.1359954)
Supplement: Supplementary file 1 [file DataSheet1.docx]

Supplementary Material

# Supplementary Table

# Table S.1 Identification of chemical components of Massa Medicata Fermentata

| No. | t_R_/min | | Name | type | Formula | | Theoretical value | | Measured value | | Secondary fragment | | MZ | | M | | S | |  |
| --- | --- | --- | --- | --- | --- | --- | --- | --- | --- | --- | --- | --- | --- | --- | --- | --- | --- | --- | --- |
| 1^a^ | 8.07 | trehalose | | Saccharides | C_12_H_22_O_11_ | | 341.1078 | | 341.1098 | | 179.0570[M-H-C_6_H_10_O_5_]^-^, 119.0357[M-H-C_6_H_10_O_5_-C_2_H_4_O_2_]^-^, 89.0248[M-H-C_6_H_10_O_5_-C_3_H_6_O_3_]^-^ | | + | |  | | + | |  |
| 2^a^ | 8.26 | maltose | | Saccharides | C_12_H_22_O_11_ | | 341.1078 | | 341.1103 | | 221.0673[M-H-C_4_H_8_O_4_]^-^, 179.0570[M-H-C_6_H_10_O_5_]^-^, 113.0251[M-H-C_6_H_10_O_5_-2H_2_O-CH_2_O]^-^ | | + | |  | | + | |  |
| 3^a^ | 8.43 | Glucuronic acid | | Saccharides | C_6_H_10_O_7_ | | 193.0343 | | 193.0363 | | 103.0042[M-H-C_3_H_6_O_3_]^-^, 97.4728[M-H-2H_2_O-C_2_H_4_O_2_]^-^, 85.0298[M-H-C_3_H_6_O_3_-H_2_O]^-^, 72.9933[M-H-C_4_H_8_O_4_]^-^ | | + | | + | |  | |  |
| 4^a^ | 8.48 | sucrose | | Saccharides | C_19_H_18_O_6_ | | 341.1020 | | 341.1096 | | 161.0466[M-H-C_6_H_10_O_5_-H_2_O]^-^, 143.0360[M-H-C_6_H_10_O_5_-2H_2_O]^-^, 119.0356[M-H-C_6_H_10_O_5_-C_2_H_4_O_2_]^-^, 113.0250[M-H-C_6_H_10_O_5_-2H_2_O-CH_2_O]^-^ | | + | |  | |  | |  |
| 5 | 8.52 | glutamate | | Amino acids | C_5_H_9_NO_4_ | | 146.0448 | | 146.0468 | | 128.0361[M-H-H_2_O]^-^, 102.0566[M-H-CO_2_]^-^ | | + | |  | |  | |  |
| 6^a^ | 8.61 | mannitol | | Saccharides | C_6_H_14_O_6_ | | 181.0707 | | 181.0725 | | 163.0619[M-H-H_2_O]^-^, 101.0249[M-H-2H_2_O-CH_2_-CH_2_O]^-^, 71.0140[M-H-2H_2_O-CH_2_-C_2_H_4_O_2_]^-^ | | + | | + | | + | |  |
| 7^a^ | 8.66 | gluconic acid | | Saccharides | C_6_H_12_O_7_ | | 195.0499 | | 195.0518 | | 177.0413[M-H-H_2_O]^-^, 159.0307[M-H-2H_2_O]^-^, 141.0201[M-H-3H_2_O]^-^, 129.0201[M-H-2H_2_O-CH_2_O]^-^, 111.0093[M-H-3H_2_O-CH_2_O]^-^ | | + | | + | | + | |  |
| 8 | 9.00 | Quinic acid | | Organic acids | | C_7_H_12_O_6_ | | 191.0550 | | 191.0569 | | 173.0100[M-H-H_2_O]^-^, 127.0409[M-H-H_2_O-HCOOH]^-^ | | + | | + | | + | |
| 9 | 9.09 | 2,4-Decadienal | | Aldehyde | | C_10_H_16_O | | 175.1093 | | 175.1078 | | 158.0812, 116.0709, 112.0760 | |  | |  | | + | |
| 10 | 9.16 | glucose | | Saccharides | | C_6_H_12_O_6_ | | 179.0550 | | 179.0570 | | 161.0463[M-H-H_2_O]-, 119.0352[M-H-C_2_H_4_O_2_]-, 113.0248[M-H-2H_2_O-CH_2_O]- | | + | | + | | + | |
| 11^a^ | 9.81 | arabinose | | Saccharides | | C_5_H_10_O_5_ | | 149.0445 | | 149.0463 | | 89.0248[M-H-C_2_H_4_O_2_]-, 71.0140[M-H-C_2_H_4_O_2_-H_2_O]-, 59.0139[M-H-C_3_H_6_O_3_]- | | + | | + | | + | |
| 12^a^ | 10.06 | malic acid | | Organic acids | | C_4_H_6_O_5_ | | 133.0131 | | 133.0150 | | 115.0043[M-H-H_2_O]- | | + | |  | |  | |
| 13^a^ | 12.84 | citric acid | | Organic acids | | C_6_H_8_O_7_ | | 191.0186 | | 191.0206 | | 173.0100[M-H-H_2_O]-, 129.02011[M-H-H_2_O-CO_2_]-, 111.0094[M-H-2H_2_O-CO_2_]- | | + | | + | | + | |
| 14^a^ | 13.76 | uridine | | Nucleotides | | C_9_H_12_N_2_O_6_ | | 243.0612 | | 243.0632 | | 200.0572[M-H-CONH]-, 110.0253[M-H-CONH-C_3_H_6_O_3_]-, 82.0301[M-H-CONH-C_3_H_6_O_3_-CO]- | | + | | + | | + | |
| 15^a^ | 13.88 | adenine | | Alkaloids | | C_5_H_5_N_5_ | | 136.0618 | | 136.0624 | | 119.0356[M+H-NH_3_]+ | | + | | + | | + | |
| 16^a^ | 14.06 | adenosine | | Nucleotides | | C_10_H_13_N_5_O_4_ | | 268.1040 | | 268.1036 | | 136.0623[M+H-rib]+ | | + | | + | | + | |
| 17^a^ | 15.48 | succinic acid | | Organic acids | | C_4_H_6_O_4_ | | 117.0182 | | 117.0200 | | 73.0297[M-H-CO_2_]- | | + | | + | | + | |
| 18^a^ | 17.89 | gallic acid | | Organic acids | | C_7_H_6_O_5_ | | 169.0131 | | 169.0149 | | 125.0251[M-H-CO_2_]- | | + | | + | | + | |
| 19 | 18.67 | protocatechuic acid | | Organic acids | | C_7_H_6_O_4_ | | 153.0182 | | 153.0179 | | 108.9893[M-H-CO_2_]- | | + | | + | |  | |
| 20^a^ | 18.93 | Phenylalanine | | Amino acids | | C_9_H_11_NO_2_ | | 164.0706 | | 164.0725 | | 147.0460[M-H-NH_3_]- | | + | | + | | + | |
| 21^a^ | 19.69 | pantothenic acid | | Vitamins | | C_9_H_17_NO_5_ | | 218.1023 | | 218.1039 | | 146.0831[M-H-C_3_H_4_O_2_]-, 88.0407[M-H-C_6_H_10_O_3_]- | | + | | + | | + | |
| 22 | 20.77 | Salvianic acid A | | Phenylpropanoids | | C_9_H_10_O_5_ | | 197.0445 | | 197.0463 | | 179.0359[M-H-H_2_O]-, 135.0459[M-H-H_2_O-CO_2_]- | | + | | + | | + | |
| 23^a^ | 20.68 | shikimic acid | | Organic acids | | C_7_H_10_O_5_ | | 173.0445 | | 173.0463 | | 93.0350[M-H-2H_2_O-CO_2_]-, 83.0505[M-H-CO_2_-HCOOH]- | |  | | + | |  | |
| 24 | 20.82 | 2,4-Decadienal | | Aldehyde | | C_12_H_20_O | | 203.1406 | | 203.1395 | | 132.1023 | |  | |  | | + | |
| 25^a^ | 21.29 | vanillic acid | | Organic acids | | C_8_H_8_O_4_ | | 167.0339 | | 167.0350 | | 123.0459[M-H-CO_2_]-, 121.0302[M-H-HCOOH]-, 105.0358[M-H-CO_2_-H_2_O]- | | + | | + | | + | |
| 26^a^ | 22.22 | Neochlorogenic acid | | Phenylpropanoids | | C_16_H_18_O_9_ | | 353.0867 | | 353.0881 | | 191.05688[M-H-C_9_H_6_O_3_]-, 179.03581[M-H-C_7_H_10_O_5_]-, 173.04636[M-H-C_9_H_6_O_3_-H_2_O]-, 135.04593[M-H-C_7_H_10_O_5_-CO_2_]- | |  | |  | | + | |
| 27 | 22.34 | 1,3,5,7-Cyclooctetraene | | Hydrocarbons | | C_8_H_8_ | | 105.0699 | | 105.0702 | | 103.0545[M+H-H_2_]+ | | + | | + | | + | |
| 28 | 23.35 | maltol | | Flavonoids | | C_6_H_6_O_3_ | | 127.0390 | | 127.0394 | | 109.0288[M+H-H_2_O]+ | | + | |  | | + | |
| 29^a^ | 23.45 | tryptophan | | Amino acids | | C_11_H_12_N_2_O_2_ | | 203.0815 | | 203.0831 | | 159.0936[M-H-CO_2_]- | | + | | + | | + | |
| 30 | 24.72 | 3,4-Dihydroxyphenylpropionic acid | | Phenylpropanoids | | C_9_H_10_O_4_ | | 181.0495 | | 181.0516 | | 163.0409[M-H-H_2_O]-, 135.0459[M-H-HCOOH]- | | + | | + | | + | |
| 31^a^ | 26.00 | 6-c-glucose-8-c-xylose apigenin | | Flavonoid glycosides | | C_27_H_30_O_15_ | | 593.1501 | | 593.1542 | | 493.1098[M-H-C_4_H_8_O_4_]-, 383.0782[M-H-C_4_H_8_O_4_-C_3_H_6_O_3_]-, 353.0676[M-H-2C_4_H_8_O_4_]-, 325.0728[M-H-2C_4_H_8_O_4_-CO]-, 297.0779[M-H-2C_4_H_8_O_4_-2CO]- | | + | | + | | + | |
| 32^a^ | 26.53 | catechin | | Flavonoids | | C_15_H_14_O_6_ | | 289.0707 | | 289.0719 | | 245.0826[M-H-CO_2_]-, 229.8705[M-H-H_2_O-C_2_H_2_O]-, 227.0702[M-H-CO_2_-H_2_O]-, 205.0510[M-H-2C_2_H_2_O]-, 203.0720[M-H-H_2_O-C_3_O_2_]-, 187.0411[M-H-CO_2_-C_3_H_6_O]-, 179.0359[M-H-C_6_H_6_O_2_]-, 165.0204[M-H-C_7_H_8_O_2_]-, 161.0615[M-H-H_2_O-C_6_H_6_O_2_]-, 137.0256[M-H-C_8_H_8_O_3_]-, 125.0251[M-H-C_9_H_8_O_3_]- | | + | |  | | + | |
| 33 | 26.83 | Phthalic acid | | Organic acids | | C_8_H_6_O_4_ | | 165.0182 | | 165.0201 | | 121.0302[M-H-CO_2_]-, 93.0349[M-H-C_2_O_3_]- | | + | | + | | + | |
| 34 | 26.85 | Caffeic acid glyceride | | Phenylpropanoids | | C_12_H_14_O_6_ | | 253.0707 | | 253.0712 | | 179.05705[M-H-C_3_H_6_O_2_]-, 161.0512[M-H-C_3_H_6_O_2_-H_2_O]-, 135.04611[M-H-C_3_H_6_O_2_- CO_2_]- | |  | |  | | + | |
| 35^a^ | 27.08 | Hydroxymethylglutaric acid | | Organic acids | | C_7_H_12_O_5_ | | 175.0601 | | 175.0620 | | 157.0515[M-H-H_2_O]-, 131.0721[M-H-CO_2_]-， 129.0566[M-H-HCOOH]-, 113.0614[M-H-H_2_O-CO_2_]-, 85.0662[M-H-CO_2_-HCOOH]- | | + | | + | | + | |
| 36^a^ | 27.47 | Schaftoside | | Flavonoid glycosides | | C_26_H_28_O_14_ | | 563.1395 | | 563.1426 | | 503.1192[M-H-C_2_H_4_O_2_]-, 473.1098[M-H-C_3_H_6_O_3_]-,  443.0995[M-H-C_4_H_8_O_4_]-, 297.0776[M-H-C_4_H_8_O_4_-C_9_H_6_O_2_]- | | + | | + | | + | |
| 37 | 27.67 | paeonol | | Phenols | | C_9_H_10_O_3_ | | 165.0546 | | 165.0563 | | 147.0461[M-H-H_2_O]-, 135.0459[M-H-CH_2_O]- | | + | | + | | + | |
| 38 | 28.38 | Protocatechuic aldehyde | | Organic acids | | C_7_H_6_O_3_ | | 137.0233 | | 137.0252 | | 109.0299[M-H-CO]-, 93.0350[M-H-CO_2_]- | | + | | + | | + | |
| 39^a^ | 28.64 | Isoschaftoside | | Flavonoid glycosides | | C_26_H_28_O_14_ | | 563.1395 | | 563.1440 | | 503.1186[M-H-C_2_H_4_O_2_]-, 473.1094[M-H-C_3_H_6_O_3_]-, 443.1001[M-H-C_4_H_8_O_4_]-, 297.0775[M-H-C_4_H_8_O_4_-C_9_H_6_O_2_]- | | + | | + | | + | |
| 40 | 28.83 | Ethyl protocatechuic acid | | Organic esters | | C_9_H_10_O_4_ | | 181.0495 | | 181.0492 | | 119.0501[M-H-CO_2_-H_2_O]- | | + | | + | |  | |
| 41^a^ | 29.14 | salicylic acid | | Organic acids | | C_7_H_6_O_3_ | | 137.0233 | | 137.0252 | | 93.0350[M-H-CO_2_]-, 65.0397[M-H-C_2_O_3_]- | | + | | + | | + | |
| 42^a^ | 29.31 | Luteoloside | | Flavonoid glycosides | | C_21_H_20_O_11_ | | 447.0922 | | 447.0957 | | 285.0410[M-H-C_6_H_10_O_5_]-, 133.0305[M-H-C_6_H_10_O_5_-C_7_H_4_O_4_]- | | + | | + | | + | |
| 43^a^ | 29.81 | 4-[2-Formyl-5- (hydroxymethyl) -1h pyrrol-1-yl] butyric acid | | Alkaloids | | C_10_H_13_NO_4_ | | 210.0761 | | 210.0778 | | 124.0411[M-H-C_4_H_6_O_2_]-, 94.0303[M-H-C_4_H_6_O_2_-CH_2_O]- | | + | | + | | + | |
| 44^a^ | 29.97 | p-hydroxybenzaldehyde | | Phenols | | C_7_H_6_O_2_ | | 121.0284 | | 121.0302 | | 93.035[M-H-CO]- | | + | | + | | + | |
| 45^a^ | 30.07 | caffeic acid | | Phenylpropanoids | | C_9_H_8_O_4_ | | 179.0339 | | 179.0362 | | 135.0459[M-H-CO_2_]-, 107.0507[M-H-C_2_O_3_]- | | + | | + | | + | |
| 46 | 30.38 | Coumaric acid glucose | | Phenylpropanoids | | C_15_H_18_O_8_ | | 325.0918 | | 325.1411 | | 163.04076[M-H-C_6_H_10_O_5_]-, 119.05085[M-H-C_6_H_10_O_5_-CO_2_]- | |  | |  | | + | |
| 47^a^ | 30.80 | Orientin | | Flavonoid glycosides | | C_21_H_20_O_11_ | | 447.0922 | | 447.0957 | | 357.0623[M-H-C_3_H_6_O_3_]-, 327.0518[M-H-C_4_H_8_O_4_]-, 299.0568[M-H-C_4_H_8_O_4_-CO]- | | + | | + | | + | |
| 48 | 31.09 | syringic acid | | Organic acids | | C_9_H_10_O_5_ | | 197.0445 | | 197.0459 | | 182.0229[M-H-CH_3_]-, 166.9993[M-H-CH_2_O]-, 123.0094[M-H-CH_2_O-CO_2_]-, 121.0304[M-H-C_2_H_4_O_3_]- | | + | | + | | + | |
| 49^a^ | 33.12 | 2-hydroxy-4-methylpentanoic acid | | Organic acids | | C_6_H_12_O_3_ | | 131.0703 | | 131.0721 | | 85.0662[M-H-HCOOH]-, 69.0348[M-H-H_2_O-C_2_H_4_O]- | | + | | + | | + | |
| 50^a^ | 33.39 | Vitexin | | Flavonoid glycosides | | C_21_H_20_O_10_ | | 431.0973 | | 431.0995 | | 341.0679[M-H-C_3_H_6_O_3_]-, 311.0564[M-H-C_4_H_8_O_4_]-, 283.0614[M-H-C_4_H_8_O_4_-CO]- | | + | | + | | + | |
| 51 | 33.48 | 3-O-Feruloylquinic acid | | Phenylpropanoids | | C_17_H_20_O_9_ | | 367.1024 | | 367.1047 | | 193.05139[M-H-C_7_H_10_O_5_]-, 173.04607[M-H-C_10_H_10_O_4_]-, 134.03810[M-H-C_7_H_10_O_5_-CO_2_-CH_3_]- | |  | |  | | + | |
| 52 | 33.84 | 5-hydroxy-7-methoxy-chromen-2-one | | Phenylpropanoids | | C_10_H_8_O_4_ | | 193.0495 | | 193.0503 | | 133.1017[M+H-CH_3_OH-CO]+ | | + | | + | | + | |
| 53 | 34.21 | Kampferol-3-O-glucoside | | Flavonoid glycosides | | C_22_H_22_O_11_ | | 461.1078 | | 461.1100 | | 371.0785[M-H-C_3_H_6_O_3_]-, 341.0679[M-C_4_H_8_O_4_]-, 298.0490[M-H-C_6_H_11_O_5_]- | | + | | + | | + | |
| 54^a^ | 34.61 | Quercetin-3-o-glucoside | | Flavonoid glycosides | | C_21_H_20_O_12_ | | 463.0871 | | 463.0891 | | 300.02783[M-H-C_6_H_11_O_5_]-, 151.00478[M-H-C_6_H_10_O_5_-C_7_H_6_O_2_-CO]- | |  | |  | | + | |
| 55 | 35.57 | 3,5-dihydroxybenzoic acid | | Organic acids | | C_7_H_6_O_4_ | | 153.0182 | | 153.0201 | | 109.0301[M-H-CO_2_]- | | + | | + | | + | |
| 56 | 36.07 | 5-hydroxy-1,4-naphthone | | Naphthoquinones | | C_10_H_8_O_3_ | | 177.0546 | | 177.0547 | | 159.1176[M+H-H_2_O]+, 149.0603[M+H-CO]+, 131.0860[M+H-H_2_O-CO]+, 121.0654[M+H-2CO]+, 103.0545[M+H-2CO-H_2_O]+ | | + | | + | |  | |
| 57 | 36.76 | Dihydroferulic acid | | Phenylpropanoids | | C_10_H_12_O_4_ | | 195.0652 | | 195.0666 | | 137.025[M-H-C_2_H_2_O_2_]- | | + | |  | | + | |
| 58 | 37.92 | L-(-)-3-Phenyllactic Acid | | Phenylpropanoids | | C_9_H_10_O_3_ | | 165.0546 | | 165.0564 | | 147.0459[M-H-H_2_O]-, 119.0508[M-H-HCOOH]-, 103.0561[M-H-H_2_O-CO_2_]- | | + | | + | | + | |
| 59^a^ | 38.00 | cinnamic acid | | Phenylpropanoids | | C_9_H_8_O_2_ | | 147.0441 | | 147.0459 | | 129.0564[M-H-H_2_O]-, 102.9493[M-H-CO_2_] | | + | | + | | + | |
| 60^a^ | 38.12 | Ferulic Acid | | Phenylpropanoids | | C_10_H_10_O_4_ | | 193.0495 | | 193.0513 | | 178.0280[M-H-CH_3_]-, 149.0616[M-H-CO_2_]-, 134.0381[M-H-CH_3_-CO_2_]- | | + | | + | | + | |
| 61^a^ | 38.50 | Scopolamine lactone | | Phenylpropanoids | | C_10_H_8_O_4_ | | 193.0495 | | 193.0502 | | 178.0267[M+H-CH_3_]+, 165.0550[M+H- | | + | | + | | + | |
| 62^a^ | 38.79 | Taxifolin | | Flavonoids | | C_15_H_12_O_7_ | | 303.0499 | | 303.0522 | | 285.0409[M-H-H_2_O]-, 275.0549[M-H-CO]-, 259.0620[M-H-CO_2_]-, 241.0511[M-H-CO_2_-H_2_O]-, 217.0515 [M-H-CO_2_-C_2_H_2_O]-, 199.0409[M-H-CO_2_-H_2_O-C_2_H_2_O]-, 125.0251[M-H-CO_2_-C_8_H_6_O_2_]- | | + | |  | |  | |
| 63 | 40.92 | kaempferol 7- rhamnopyranoside | | Flavonoid glycosides | | C_21_H_20_O_10_ | | 431.0973 | | 431.0990 | | 285.0407[M-H-C_6_H_10_O_4_]-, 257.0457[M-H-C_6_H_10_O_4_-CO]- | | + | | + | |  | |
| 64^a^ | 41.57 | azelaic acid | | Organic acids | | C_9_H_16_O_4_ | | 187.0965 | | 187.0984 | | 169.0877[M-H-H_2_O]-, 125.0979[M-H-H_2_O-CO_2_]-, 123.0823[M-H-H_2_O-HCOOH]- | | + | | + | | + | |
| 65^a^ | 42.63 | p-coumaric acid | | Phenylpropanoids | | C_9_H_8_O_3_ | | 163.0390 | | 163.0408 | | 119.0509[M-H-CO_2_]- | | + | | + | | + | |
| 66^a^ | 43.90 | Luteolin | | Flavonoids | | C_15_H_10_O_6_ | | 285.0394 | | 285.0406 | | 199.0408[M-H-C_4_H_6_O_2_]-, 175.0409[M-H-C_6_H_6_O_2_]-, 151.0045[M-H-C_8_H_6_O_2_]-, 133.0302[M-H-C_7_H_4_O_4_]- | | + | | + | | + | |
| 67 | 43.98 | β-ionone | | Terpenes | | C_13_H_20_O | | 193.1587 | | 193.1594 | | 149.1331[M+H-C_2_H_4_O]+, 133.1017[M+H-C_3_H_8_O]+, 109.1014[M+H-C_5_H_8_O]+ | |  | | + | |  | |
| 68^a^ | 44.19 | quercetin | | Flavonoids | | C_15_H_10_O_7_ | | 301.0343 | | 301.0359 | | 273.0401[M-H-CO]-, 178.9994[M-H-C_7_H_6_O_2_]-, 151.0045[M-H-C_7_H_6_O_2_-CO]- | | + | | + | | + | |
| 69^a^ | 44.53 | isorhamnetin | | Flavonoids | | C_16_H_12_O_7_ | | 315.0499 | | 315.0504 | | 300.0280[M-H-CH_3_]-, 271.0298[M-H-CO_2_]- | | + | | + | | + | |
| 70^a^ | 44.65 | Trihydroxyoctadecadienoic acid | | Organic acids | | C_18_H_32_O_5_ | | 327.2166 | | 327.2183 | | 229.1450[M-H-C_6_H_10_O]-, 171.1034[M-H-C_9_H_16_O_2_]-, 127.1133[M-H-C_9_H_16_O_2_-CO_2_]- | | + | | + | | + | |
| 71^a^ | 45.28 | 9(S),12(S),13(S)-Trihydroxy-10(E)-octadecenoicAcidor 9,10,11-trihydroxy-12-octadecaenoic acid | | Organic acids | | C_18_H_34_O_5_ | | 329.2323 | | 329.2341 | | 229.1448[M-H-C_6_H_12_O]-, 211.1345[M-H-C_6_H_14_O_2_]-, 171.1035[M-H-C_9_H_18_O_2_]- | | + | | + | | + | |
| 72^a^ | 45.31 | apigenin | | Flavonoids | | C_15_H_10_O_5_ | | 269.0444 | | 269.0455 | | 225.0562[M-H-CO_2_]-, 117.0352[M-H-C_7_H_4_O_4_]- | | + | | + | | + | |
| 73^a^ | 45.68 | Chrysoeriol | | Flavonoids | | C_16_H_12_O_6_ | | 299.0550 | | 299.0569 | | 284.0329[M-H-CH_3_]-, 256.0379[M-H-CH_3_-CO]- | | + | | + | | + | |
| 74 | 45.83 | 6-Methoxyluteolin | | Flavonoids | | C_16_H_12_O_7_ | | 315.0499 | | 315.0514 | | 300.028[M-H-CH_3_]- | | + | |  | | + | |
| 75 | 45.87 | 2',6'-dihydroxy -4,4'-Dimethoxychalcone | | Flavonoids | | C_17_H_16_O_5_ | | 299.0914 | | 299.0936 | | 181.06699[M-H-C_7_H_2_O_2_]- | |  | |  | | + | |
| 76^a^ | 46.91 | Genistin | | Flavonoid glycosides | | C_21_H_20_O_10_ | | 433.1129 | | 433.1129 | | 271.0617[M+H-C_6_H_10_O_6_]+ | |  | | + | |  | |
| 77 | 46.95 | 6-Hydroxycoumarinor its isomers | | Phenylpropanoids | | C_9_H_6_O_3_ | | 163.0390 | | 163.0394 | | 145.1014[M+H-H_2_O]+, 135.0444[M+H-CO]+, 117.0704[M+H-H_2_O-CO]+ | | + | | + | |  | |
| 78 | 47.46 | N-Stearylethanolamine | | Amides | | C_20_H_41_NO_2_ | | 328.3210 | | 328.3204 | | 310.3096[M+H-H_2_O]+ | | + | | + | |  | |
| 79^a^ | 48.16 | Isobutyl 4-hydroxybenzoate | | Organic esters | | C_11_H_14_O_3_ | | 193.0859 | | 193.0878 | | 137.0253[M-H-C_4_H_8_]- | | + | | + | | + | |
| 80^a^ | 48.41 | Octadecatrien-4-keto acid | | Organic acids | | C_18_H_28_O_3_ | | 293.2111 | | 293.2110 | | 275.2000[M+H-H_2_O]+, 149.0965[M+H-H_2_O-C_8_H_14_O]+, 107.0858[M+H-H_2_O-C_11_H_20_O]+ | | + | | + | | + | |
| 81 | 48.49 | ethyl linoleate | | Organic acids | | C_20_H_36_O_2_ | | 309.2788 | | 309.2783 | | 277.2153[M+H-2CH_4_]+ | | + | |  | | + | |
| 82 | 48.75 | vitexicarpin | | Flavonoids | | C_19_H_18_O_8_ | | 373.0918 | | 373.0931 | | 358.0705[M-H-CH_3_]-, 343.0472[M-H-2CH_3_]-, 328.0233[M-H-3CH_3_]-, 300.0282[M-H-3CH_3_-CO]- | | + | | + | | + | |
| 83^a^ | 49.10 | (9z, 11e) -13-hydroperoxyoctadecane-9,11-dienoic acid | | Organic acids | | C_18_H_32_O_4_ | | 311.2217 | | 311.2231 | | 293.2121[M-H-H_2_O]-, 249.2233[M-H-H_2_O-CO_2_]- | | + | | + | | + | |
| 84 | 49.57 | 3,5-Dihydroxy hexadecane acid | | Organic acids | | C_16_H_32_O_4_ | | 287.2217 | | 287.2230 | | 269.2117[M-H-H_2_O]-, 241.2179[M-H-HCOOH]- | | + | | + | | + | |
| 85 | 49.88 | 3,5,6,7,8,3 ', 4' - heptamethoxyflavonoid | | Flavonoids | | C_22_H_24_O_9_ | | 433.1493 | | 433.1521 | | 417.1192[M+H-CH_4_]+, 403.1020[M+H-2CH_3_]+, 388.0791[M+H-3CH_3_]+ | |  | | + | |  | |
| 86^a^ | 50.31 | (9z) -12,13-dihydroxyoctadecan-9-enoic acid | | Organic acids | | C_18_H_34_O_4_ | | 313.2373 | | 313.2386 | | 183.1398[M-H-C_7_H_14_O_2_]-, 129.0928[M-H-C_11_H_20_O_2_]-, 99.0819[M-H-C_11_H_20_O_2_-CH_2_O]- | | + | | + | | + | |
| 87 | 50.76 | 5-hydroxy-6,7,8,3 ', 4' - pentamethoxyflavone | | Flavonoids | | C_20_H_20_O_8_ | | 389.1231 | | 389.1227 | | 374.0991[M+H-CH_3_]+, 341.0649[M+H-2CH_3_-H_2_O]+, 331.0807[M+H-2CH_3_-CO]+ | | + | | + | | + | |
| 88 | 51.17 | octadecatetraenoic acid | | Organic acids | | C_18_H_28_O_2_ | | 277.2162 | | 277.2156 | | 93.0702[M+H-C_11_H_20_O_2_]+ | | + | | + | | + | |
| 89^a^ | 53.11 | 9-hydroxy-10,12-octadecadienoic acid | | Organic acids | | C_18_H_32_O_3_ | | 295.2268 | | 295.2285 | | 277.2176[M-H-H_2_O]-, 195.1404[M-H-C_6_H_12_O]-, 171.1035[M-H-C_9_H_16_]- | | + | | + | | + | |
| 90 | 53.27 | linolenic acid | | Organic acids | | C_18_H_30_O_2_ | | 279.2319 | | 279.2321 | | 147.1181, 123.1174, 109.1016, 137.1331, 135.1173 | | + | | + | | + | |
| 91 | 54.23 | linoleic acid | | Organic acids | | C_18_H_32_O_2_ | | 279.2319 | | 279.2332 | | 261.2229[M-H-H_2_O]- | | + | |  | |  | |
| 92 | 54.37 | Methoxypentadecanoic acid | | Organic acids | | C_16_H_32_O_3_ | | 271.2268 | | 271.2277 | | 253.2171[M-H-H_2_O]-, 225.2229[M-H-HCOOH]- | | + | | + | | + | |
| 93 | 54.66 | palmitamide | | Amides | | C_16_H_33_NO | | 256.2635 | | 256.2634 | | 102.0918, 88.0759 | | + | |  | | + | |
| 94^a^ | 55.27 | phthalic anhydride | | Anhydrides | | C_8_H_4_O_3_ | | 149.0233 | | 149.0238 | | 121.0287[M+H-CO]+ | | + | | + | |  | |
| 95 | 57.62 | 12-hydroxyoctadecanoic acid | | Organic acids | | C_18_H_36_O_3_ | | 299.2581 | | 299.2597 | | 281.2477[M-H-H_2_O]-, 253.2544[M-H-HCOOH]- | | + | | + | | + | |

# Note: MZ: natural fermented MMF not inoculated with Rhizopus oryzae; M: Rhizopus oryzae inoculation fermentation MMF; S: commercial MMF A; a: represents comparison and identification with secondary mass spectrometry data in MassBank database; +: indicates that the component is detected.
